# Synthesis and Properties of Octithiophene Dication Sterically Segregated by Annelation with Bicyclo[2.2.2]octene Units

**DOI:** 10.3390/ma3032037

**Published:** 2010-03-19

**Authors:** Tohru Nishinaga, Daisuke Yamazaki, Masaki Tateno, Masahiko Iyoda, Koichi Komatsu

**Affiliations:** 1 Department of Chemistry, Graduate School of Science and Engineering, Tokyo Metropolitan University, Hachioji, Tokyo 192-0397, Japan; E-Mails: tateno-masaki@ed.tmu.ac.jp (M.T.); iyoda@tmu.ac.jp (M.I.); 2 Institute for Chemical Research, Kyoto University, Uji, Kyoto 611-0011, Japan; E-Mails: yamazaki.daisuke@me.m-kagaku.co.jp (D.Y.); komatsu@fukui-ut.ac.jp (K.K.)

**Keywords:** bipolaron, dication, oligothiophene, polaron pair, singlet biradical

## Abstract

Octithiophene sterically segregated by annelation with bicyclo[2.2.2]octene (BCO) units was synthesized to study the unimolecular properties of longer oligothiophene dications. For the preparation of such longer oligomers, a new route for the synthesis of the monomer annelated with BCO unit at the 3,4-positions of thiophene ring was developed. Attempted synthesis of octithiophene **1**(8T) fully annelated with BCO units was hampered by low solubility of the product, and octithiophene **2**(8T) having octyl groups instead of the BCO units at the second rings from the both ends of **1**(8T) was synthesized to solve the solubility problem. Neutral **2**(8T) has a lower planarity due to the steric repulsion between the octyl substituents and the neighboring thiophene units. However, dication **2**(8T)^2+^, which was obtained as a stable salt by chemical two-electron oxidation with NO^+^SbF_6_^–^, has a planar quinoid structure, as judged from a linear correlation between the inverse chain length and the absorption energy for **1**(nT)^2+^ (n = 3,4,6) and **2**(8T)^2+^. Based on the comparison with the calculated absorption spectra and the result of ESR inactive properties, **2**(8T)^2+^ appears to have a singlet ground state with open-shell biradical character rather than a closed-shell singlet structure.

## 1. Introduction

Polythiophenes are one of the most useful conducting polymers, and their conduction mechanism is a subject of intense theoretical and experimental studies [1,2,3,4,5]. Nevertheless, the fundamental electronic structure of doped-states of the single π-conjugated chain has been elusive. The low spin character of polythiophenes at high p-doping level was originally rationalized by the physical terms of bipolaron, which corresponds to closed-shell singlet dication in the chemical terms [6]. However, the investigations into various oligothiophenes as models of polythiophenes revealed that intra- and inter-chain polaron pairs, which correspond to singlet biradical dication [7] and radical cation π-dimer [8], respectively, can be alternatives to bipolaron. So far, a few radical cation π-dimers of shorter oligothiophenes have been unambiguously isolated and characterized by X-ray crystallography [9,10,11], but the existence of such inter-chain interaction between longer oligomer dications is under debate.

In 1998, Janssen *et al*. reported that the absorption spectrum of dodecithiophene (12T) dication with dodecyl side chains in CH_2_Cl_2_ solution was incompatible with those of the corresponding shorter hexamer (6T) and nonamer (9T) dications [7]. By combining this with the results of ESR measurements and semi-empirical theoretical calculations, they concluded that the absorption spectrum of the dication of 12T was assigned as an intra-chain polaron pair (singlet biradical dication). Recent density functional theory (DFT) studies supported the singlet biradical character of longer oligothiophene dications [12,13], although the theoretical studies suggested that not only 12T^2+^, but also 6T^2+^ and 9T^2+^, have a biradical character. However, other recent experimental studies by Otsubo and Aso groups exhibited that the absorption spectra of the dications of sterically hindered 12T in CH_2_Cl_2_ solution are totally different from those of the dications of sterically unhindered 12T [14,15]. From these results, they suggested that the π-dimer is formed in the dications of sterically unhindered 12T, while the electronic structure of the dications of sterically hindered 12T in solution can be assigned as bipolaron (closed-shell singlet dication).

As our approach to address the issue, we designed oligothiophenes **1**(nT) (n = 2, 3, 4, 6) fully annelated with bicyclo[2.2.2]octene (abbreviated as BCO) [16,17]. This structural modification was expected to not only inhibit the possible intermolecular interaction [18], which would make the interpretation of observed spectra complex, but also to be quite effective for stabilization of the cationic π-systems [19]. Thus, we succeeded in the first systematic X-ray structural analysis of cationic oligothiophenes, *i.e.,* radical cation and dication salts of **1**(nT) (n = 2, 3, 4, 6) [17]. These results unambiguously demonstrated that the cationic oligothiophenes are sterically segregated and hence serves as good models of single polythiophene chain at p-doped states. Using these model systems, quinoidal character of cationic oligothiophenes was shown based on the experimental results of X-ray crystallography and UV-vis-NIR, ESR, and NMR spectroscopies together with the results of DFT calculations. Then, we attempted to synthesize octamer **1**(8T) to examine the detailed electronic structure of longer oligomer dication, but unexpected low solubility of **1**(8T) hampered the further study. Therefore, we designed and synthesized **2**(8T), in which two BCO units were replaced by octyl groups, to solve the solubility problem, and conducted CV, UV-vis-NIR, and ESR measurements of the neutral and cationic species as a part of a series of studies on sterically segregated oligothiophenes by the BCO-annelation. Here we report these results and compare them with the previous results [16,17] for shorter chain homologs **1**(nT) (n = 2, 3, 4, 6).

**Figure 1:**
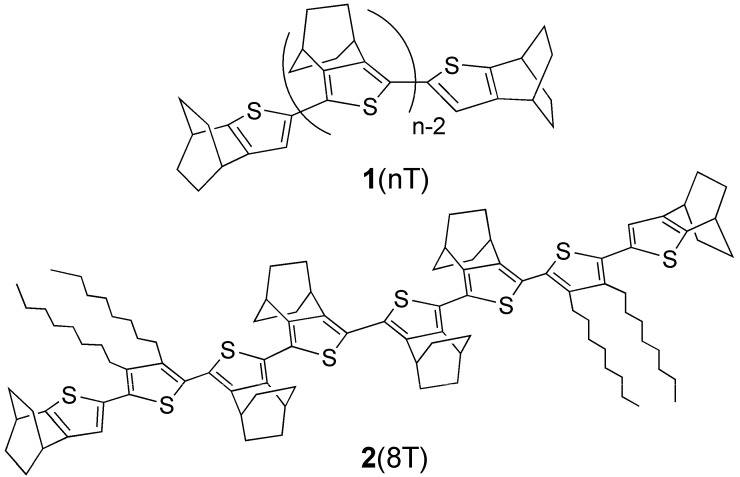
Chemical structures of **1**(nT) and **2**(8T).

## 2. Results and Discussion

### 2.1. Synthesis

**Scheme 1:**
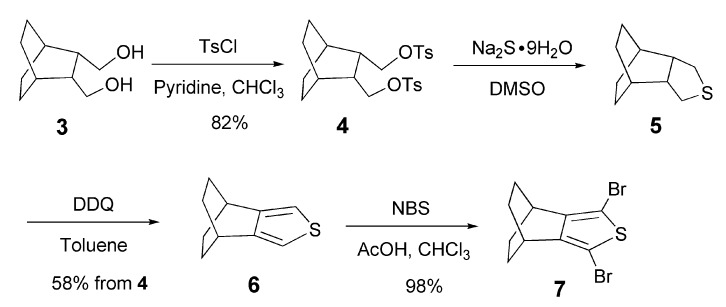
Synthesis of monomer **6** and its dibromination.

For the synthesis of longer oligothiophenes bearing BCO units, monomer** 6 **annelated with BCO at the 3,4-positions of a thiophene ring is indispensable. However, our previous route to **6** [16] involved the preparation of bicyclo[2.2.2]octane-2,3-dione [20], which required the use of a large amount of bothersome potassium metal. In addition, the yields of both the thiophene synthesis (37%) by Hinsberg method from the 2,3-dione and the following bromination-decarboxylation (42%) were moderate. Thus, we developed a new route for the synthesis of **6** as shown in Scheme 1. *cis*-2,3-Bis(hydroxymethyl)bicyclo[2.2.2]octane (**3**) [21], which is easily prepared by Diels-Alder reaction of cyclohexadiene with maleic anhydride followed by reduction with LAH and then hydrogenation, was tosylated to give **4** [22]. The reaction of **4** with sodium sulfide [22], followed by aromatization with DDQ afforded thiophene **6**. Also dibrominatation of **6** with 2 equivalents of NBS gave **7** in good yield.

**Scheme 2:**
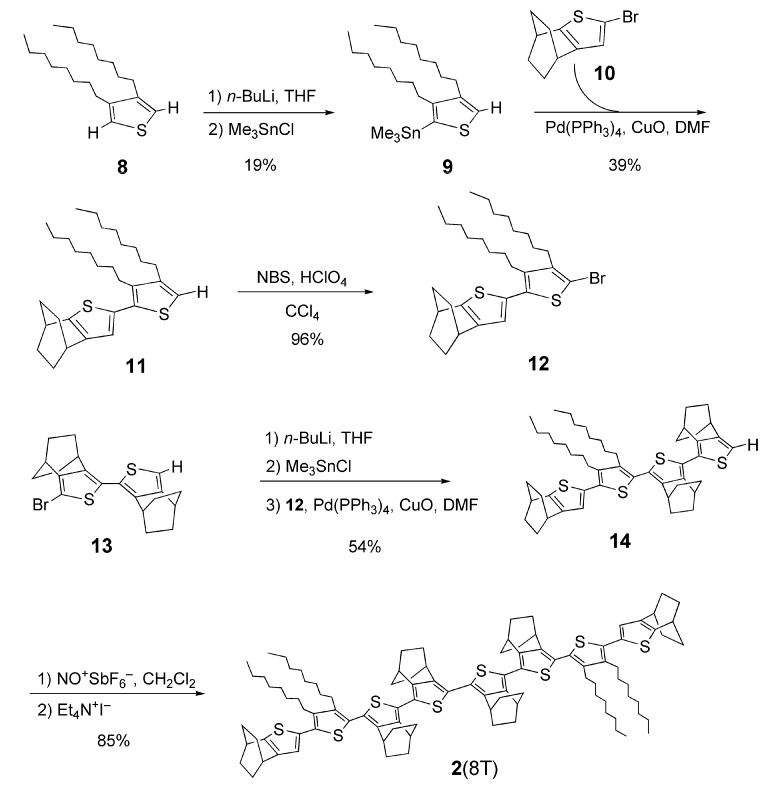
Synthesis of **2**(8T).

The attempted synthesis of octithiophene **1** (8T) by oxidative dimerization of the corresponding quaterthiophene in the same way as for **1**(6T) [16] failed due to the solubility problem of **1** (8T). Thus, octithiophene **2** (8T) was synthesized as shown in Scheme 2. Stannylated thiophene having octyl substituents **9** was subjected to Stille coupling reaction with 2-bromo-4,5-bicyclo[2.2.2]octenothiophene (**10**) to give bithiophene **11**. Bromination of **11** followed by Stille coupling with stannylated bithiophene prepared from **13** [16] gave quaterthiophene **14** in 54% yield. Then, the oxidative dimerization of **14** afforded octithiophene **2**(8T) as yellow solid in 85% yield. Octithiophene **2**(8T) was found to dissolve in most of organic solvents in satisfactory concentration.

### 2.2. UV-vis spectra and cyclic voltammetry of neutral oligothiophenes

**Figure 2:**
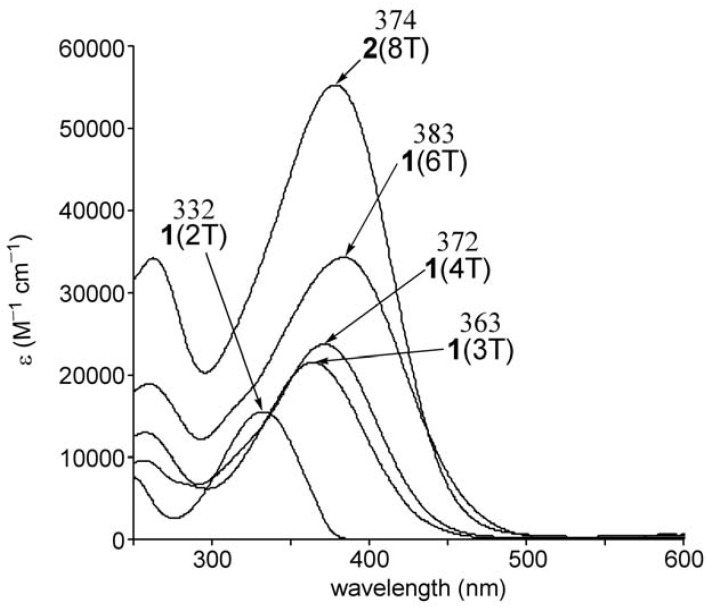
UV-vis spectra of **1**(nT) (n = 2, 3, 4, 6) and 2(8T).

The electronic absorption spectra of a series of BCO-annelated oligothiophenes **1**(2T–6T) [16] and **2**(8T) in CH_2_Cl_2_ are shown in Figure 2. The longest wavelength absorptions of **1** ascribed to the HOMO-LUMO transition are bathochromically shifted with a concomitant increase in absorption intensity, even though **1** has a distorted structure due to the steric repulsion between the bridgehead hydrogen atom of the BCO units and the adjacent thiophene rings [16]. The longest wavelength absorption of **2**(8T) having octyl groups appeared at the even shorter wavelength than that of **1**(6T) in spite of its longer π-system. This can be attributed to the further decrease of planarity of the π-system due to the steric repulsion between octyl groups at the β,β’-positions and the neighboring thiophene units. In comparison, the maximum (439 nm) of the longest wavelength absorption of octithiophene having four dodecyl substituents [23] is 65 nm longer than that of **2**(8T).

**Figure 3:**
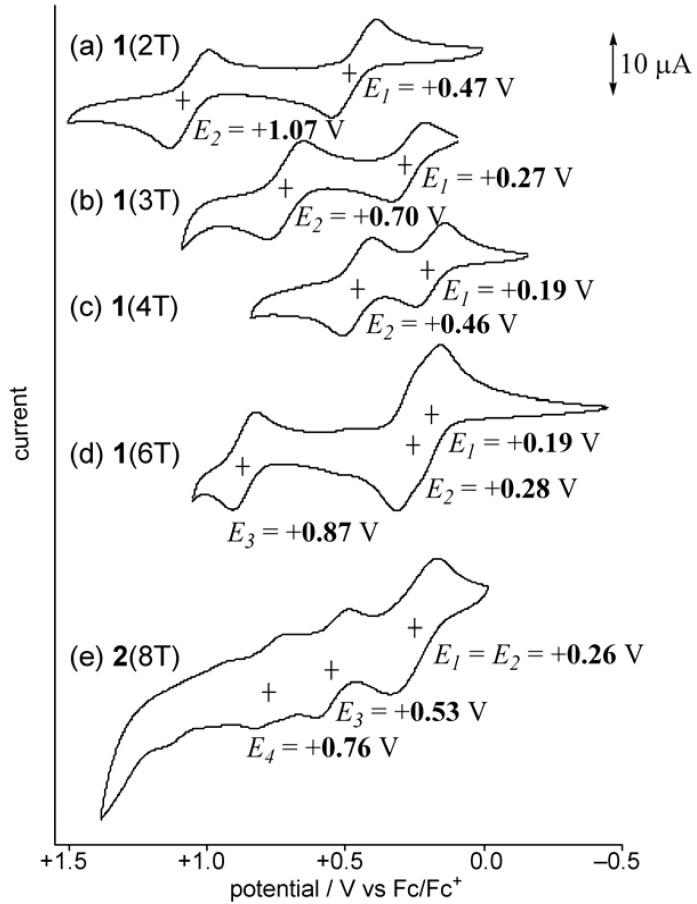
Cyclic voltammograms of **1**(nT) (n = 2, 3, 4, 6) and **2**(8T) in CH_2_Cl_2_ containing TBAPF_6_ (0.1M) with scan rate 0.1 V s^−1^: (a) **1**(2T) at −78 °C under vacuum, (b) **1**(3T) at room temperature under vacuum, (c) **1**(4T) at room temperature, (d) **1**(6T) at room temperature, and (d) **2**(8T) at room temperature.

To examine the redox behavior of **2**(8T), the cyclic voltammetry was conducted in CH_2_Cl_2_ at room temperature. The voltammogram is shown in Figure 3, together with those for **1**(2T–6T) [16]. All the oligothiophenes **1**(2T–6T) and** 2**(8T) exhibited reversible first and second oxidation waves at room temperature, except for the second oxidation wave of **1**(2T), which was irreversible at room temperature but became reversible at –78 °C [16]. As for octamer **2**(8T), the first and second oxidation waves were overlapped and observed at *E*_1/2_ = +0.26 V as a two-electron oxidation process. In addition, the third and fourth oxidation waves were observed at *E*_1/2_ = +0.53 V and *E*_1/2_ = +0.76 V, respectively, suggesting the possibility of the generation of radical trication and tetracation.

**Figure 4:**
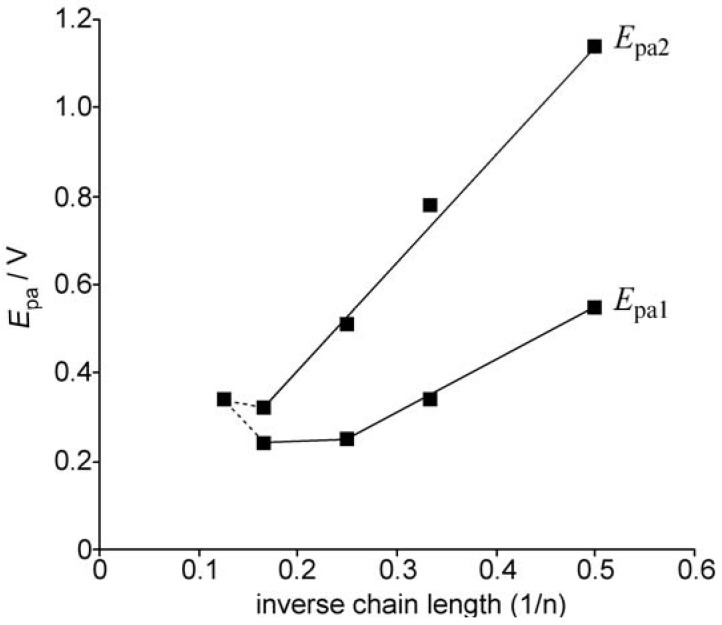
Correlation of the first (*E*_pa1_) and second oxidation peak potentials (*E*_pa2_) of the oligothiophene **1**(nT) (n = 2, 3, 4, 6) and **2**(8T) with the inverse chain length (1/n).

The correlations of the first and second oxidation potentials of oligothiophenes **1** and **2** with the inverse chain length were examined, and the results are shown in Figure 4. The oxidation potential of **1** was gradually lowered with increasing chain length. However, a saturation phenomenon was observed for the first oxidation potential as the size of oligothiophene reached tetramer **1**(4T), and no further lowering of the oxidation potential was observed for **1**(6T) [16]. In contrast, the relationship was linear for the second oxidation potential in the range of **1**(2T) to **1**(6T) so that the difference in oxidation potential (Δ(*E*_1_–*E*_2_)) becomes quite small in the case of **1**(6T). For the octamer **2**(8T), the Δ(*E*_1_–*E*_2_) value is finally observed to reach zero, although the substitution pattern is different from that of the series of **1**.

These observations would reflect the following two factors. First, the steric repulsion between the octyl groups as well as bridgehead hydrogen of the BCO unit and the adjacent thiophene rings causes a distortion in the π-system, which brings about a positive shift in the *E*_pa1_ of **2**(8T). Secondly, in spite of such unfavorable steric repulsion against the planarity of the π-system, the formation of radical cation by one-electron oxidation tends to bring about planarizations due to the formation of a quinoid structure, and the effective conjugation length is increased so that the Δ(*E*_1_–*E*_2_) value becomes zero for **2**(8T).

### 2.3. Preparation of a dication salt of **2**(8T) and its properties

**Scheme 3:**
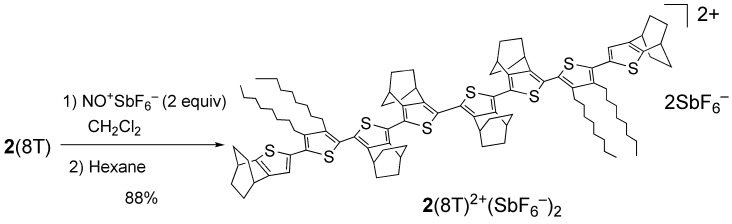
Synthesis of **2**(8T)^2+^(SbF_6_^–^)_2_.

In place of hardly soluble **1**(8T), octithiophene **2**(8T) was subjected to oxidation reaction as shown in Scheme 3. A yellow solution of **2**(8T) in CH_2_Cl_2_ was treated with two equivalents of NO^+^SbF_6_^–^ to cause immediate dark blue coloration of the solution. Then, recrystallization by slow diffusion of hexane to this solution gave a dark blue solid. Elemental analysis suggested that this solid is the dication salt, **2**(8T)^2+^(SbF_6_^–^)_2_, although the experimental error was large (–2.44% for C, –0.14% for H). The ^1^H NMR spectrum of this blue solid in CD_2_Cl_2_ exhibited broad signals, which was considered to be caused by the open-shell biradical character of **2**(8T)^2+^ and/or the fast exchange of electron between **2**(8T)^2+^ and the corresponding radical cation as a contaminant.

**Figure 5:**
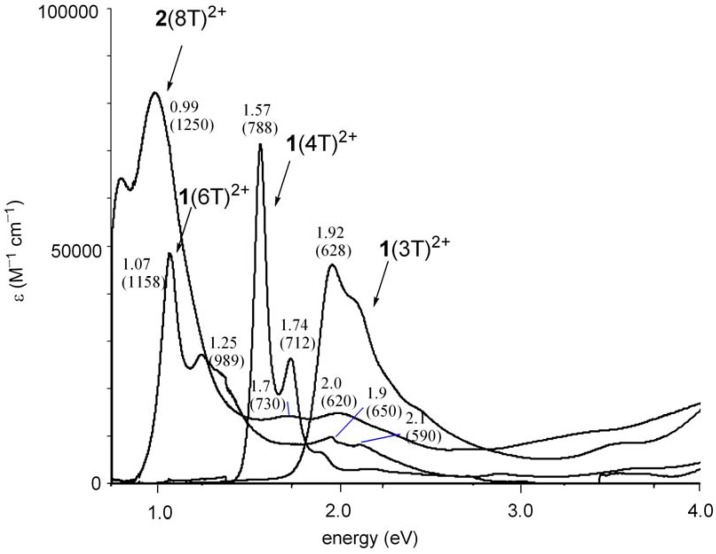
UV-vis-NIR spectra of **1**(nT)^2+^ (n = 3, 4, 6) and **2**(8T)^2+^. The numbers in parentheses are the absorptions in wavelength scale (nm).

**Figure 6:**
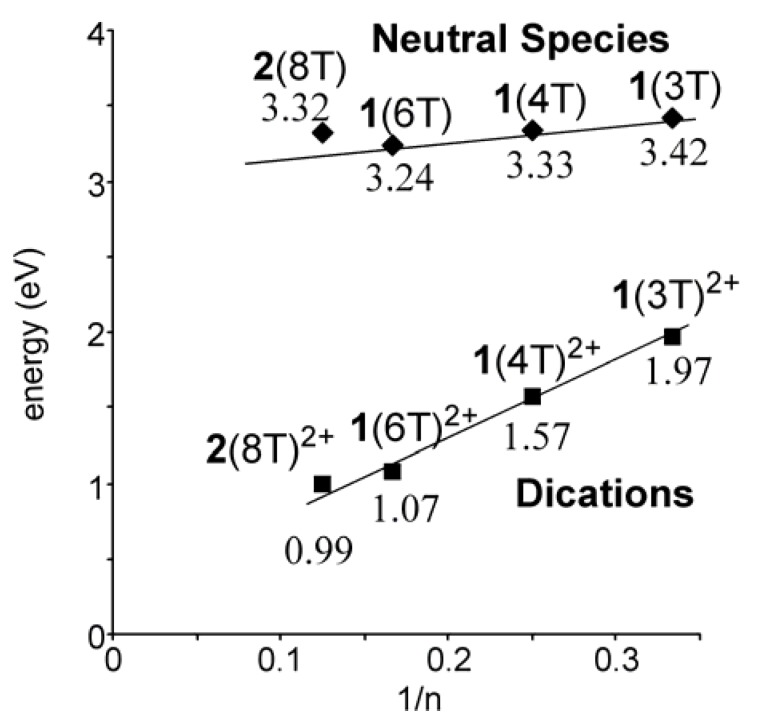
Correlation of the longest wavelength absorption of **1**(nT) (n = 3, 4, 6), **2**(8T), **1**(nT)^2+^ (n = 3, 4, 6) and **2**(8T)^2+^.

The UV-vis-NIR spectra of the dication **1**(nT)^2+^ (n = 3, 4, 6) and **2**(8T)^2+^ dissolved in CH_2_Cl_2_ are shown in Figure 5. The absorption maximum showed bathochromic shifts with increasing chain length, in accord with the expansion of π-conjugation of the oligothiophene π-system. This is confirmed by a linear correlation between the lowest absorption energy (eV) of the dications and the inverse chain length (1/n) as shown in Figure 6.

For neutral **1**(nT) (n = 3, 4, 6) a linear correlation was observed [16], but the absorption energy of **2**(8T), having octyl side chains, deviated from the linearity because of a larger twisting in the oligothiophene due to the aforementioned steric repulsion by octyl groups. In contrast, the linear correlation in the dications including **2**(8T)^2+^ implies that the steric repulsion by octyl groups hardly affects the planarity and properties of the π-conjugated system in the case of dications. A regression analysis of the linear correlation (axis intercept, 0.32; slope, 4.93; correlation coefficient 0.987) demonstrated that the predicted longest-wavelength absorption of the infinite chain is 0.32 eV. The difference in planarity between neutral and dicationic states appears to result from the difference in the electronic structures. The contribution of the quinoidal structure in **2**(8T)^2+^ is considered to overcome the force to distort the π-system by steric repulsion.

As concerns the detailed electronic structure of oligothiophene dications (nT^2+^), recent DFT calculations demonstrated that polaron pair (singlet biradical) is the ground state of longer oligomer dications [12,13]. Also, time-dependent (TD) DFT calculations (TDB3P86-30%) predicted that the singlet biradical state of quater-, sexi- or octi-thiophene dications (4T^2+^, 6T^2+^, 8T^2+^) have three or four absorption bands in contrast to one strong absorption band accompanied by one weak absorption for the closed-shell singlet states [5]. Furthermore, the calculated oscillator strengths of the closed-shell singlet states were predicted to increase with increasing chain length, and the values were larger than that of the open-shell singlet biradicals with same chain length (Table 1) [5]. Here we estimated biradical index of 8T^2+^ by natural orbital occupancy in LUMO [24,25,26] using broken-symmetry (UB3LYP/6-31G*) method and the result was 46%. Judging from these predictions, the absorption spectra of **1**(6T)^2+^ and **2**(8T)^2+^ appear to be assigned as open-shell biradical rather than the closed-shell species because **1**(6T)^2+^ and **2**(8T)^2+^ show four and three absorption bands (**1**(6T)^2+^: 1.07, 1.25, 1.9, 2.1 eV; **2**(8T)^2+^: 0.99, 1.7, 2.0 eV) and because the absorption coefficient of the lowest energy band for **1**(6T)^2+^ is smaller than that for **1**(4T)^2+^. In addition to these results from absorption spectra, variable temperature ESR measurement of dication salt **2**(8T)^2+^(SbF_6_^–^)_2_ in CH_2_Cl_2_ solution revealed that an observed very weak signal (*g* = 2.0024, peak-to-peak width 0.28 mT) is due to a radical impurity rather than the thermally excited triplet state. Overall, **2**(8T)^2+^ as well as **1**(6T)^2+^ is considered to be singlet ground state probably with an open-shell biradical character.

**Table 1:** Excitation energy in eV of closed-shell 3T^2+^, 4T^2+^, 6T^2+^ and 8T^2+^ and open-shell 4T^2+^, 6T^2+^ and 8T^2+^ at the TDB3P86-30% level (oscillator strength in parentheses) [5].

Molecule	State	E1	E2	E3	E4
3T^2+^	closed-shell	2.62 (0.68)	3.02 (0.56)		
4T^2+^	closed-shell	2.19 (1.62)	2.97 (0.21)		
	open-shell	1.88 (0.37)	2.24 (1.11)	3.04 (0.15)	
6T^2+^	closed-shell	1.59 (2.78)	2.98 (0.16)		
	open-shell	1.40 (1.44)	1.80 (0.87)	2.09 (0.10)	2.23 (0.26)
8T^2+^	closed-shell	1.22 (3.69)	2.67 (0.17)		
	open-shell	1.10 (2.01)	1.57 (0.64)	1.87 (0.93)	

## 3. Conclusions

As a series of sterically segregated oligothiophenes, octithiophene **2**(8T) was newly synthesized, and its dication salt **2**(8T)^2+^(SbF_6_^–^)_2_ was obtained by chemical two-electron oxidation with NO^+^SbF_6_^–^. Although the bulky octyl groups cause twisting of the oligothiophene chain, the dicationic state was shown to be planarized owing to the contribution of a quinoidal structure. From the comparison of the observed absorption spectra with the calculated ones and based on the result of ESR inactive properties, it was suggested that **2**(8T)^2+^ as well as **1**(6T)^2+^ have singlet ground state with open-shell biradical character rather than a closed-shell singlet structure, similarly to the case of the neutral oligothiophenes with a quinoidal structure [27,28,29].

## 4. Experimental Section

### 4.1. General

^1^H (300 or 500 MHz) and ^13^C (75.4 or 125 MHz) NMR spectra were recorded on a Varian Mercury-300 or JEOL L-500 spectrometer. Chemical shifts were reported in ppm with reference to tetramethylsilane, using the signal of solvents as internal standard (δ 7.26 or 5.32 for CHCl_3_ or CDHCl_2_ in ^1^H NMR and δ 77.0 or 54.0 in ^13^C NMR for CDCl_3_ or CD_2_Cl_2_, respectively). UV-vis-NIR spectra were recorded on a Shimadzu UV-3150 spectrometer. Cyclic voltammetry (CV) was performed on a BAS-100W electrochemical analyzer. The CV cell consisted of a glassy carbon working electrode, a Pt wire counter electrode, and a Ag/AgNO_3_ reference electrode. The measurements were carried out in 1.0 mM solutions of substrate using tetrabutylammonium hexafluorophosphate (TBAPF_6_) as a supporting electrolyte (0.1 M), and the oxidation potential values were calibrated with ferrocene. ESR spectra were recorded on a Bruker EMX spectrometer. Preparative gel-permeation chromatography (GPC) was performed with a JAI LC-908 or LC-918 chromatograph equipped with JAIGEL 1H and 2H columns. APCI mass spectra were obtained on a Finigan TSQ7000 spectrometer. High- and low-resolution EI and FAB mass spectra were obtained on a JEOL JMS-700 spectrometer.

*cis*-2,3-Bis(hydroxymethyl)bicyclo[2.2.2]octane (**3**) [21,22], 3,4-dioctylthiophene (**8**) [30], 2-bromo-4,5-bicyclo[2.2.2]octenothiophene (**10**) [16], and 5-bromo-3,4:3',4'-bis(bicyclo[2.2.2]octano)-2,2'-bithiophene (**13**) [16] were prepared according to the literature procedures.

All calculations were performed with the Gaussian 03 program [31]. The geometry optimizations and estimation of singlet biradical character was performed using a symmetry-broken UB3LYP/6-31G* method and natural orbital analysis.

### 4.2. cis-2,3-Bis(tosyloxymethyl)bicyclo[2.2.2]octane *(**4**)*

A solution of diol **3** (7.40 g, 43.5 mmol) in CHCl_3_ (90 mL) was added dropwise to a stirred solution of tosyl chloride (33.3 g, 175 mmol) in pyridine (90 mL) at 0 °C under nitrogen. After stirring at 0 °C for 30 min and at room temperature for 2 h, the reaction mixture was left to stand in a refrigerator overnight. The mixture was poured into ice-cold hydrochloric acid, extracted with CHCl_3_, washed with aqueous NaHCO_3_ and brine, and dried over Na_2_SO_4_. The volatiles were removed in vacuo, and the residue was purified by column chromatography (SiO_2_) eluted with CHCl_3_ to give ditosylate **4** (17.0 g, 35.6 mmol, 82%) as a colorless solid: ^1^H NMR (CDCl_3_) *δ* 7.77 (d, 4H, *J* = 8.2 Hz), 7.37 (d, 4H, *J* = 8.2 Hz), 4.11–4.08 (m, 2H), 3.96–3.92 (m, 2H), 2.47 (s, 6H), 2.15–2.11 (m, 2H), 1.59–1.57 (m, 2H), 1.56–1.52 (m, 2H), 1.46–1.43 (m, 2H), 1.36–1.28 (m, 4H); ^13^C NMR (CDCl_3_) *δ* 144.88, 132.67, 129.90, 127.81, 69.41, 36.82, 25.71, 25.57, 21.62, 19.99; EI-MS: *m/z* = 307 (M^+^–OTs).

### 4.3. 3,4-bicyclo[2.2.2]octanothiophene *(**6**)*

To a mixture of **4** (8.55 g, 17.9 mmol) and Na_2_S·9H_2_O (9.45 g, 39.3 mmol) under nitrogen, DMSO (90mL) was added and the solution was heated at 130 °C overnight. After cooling to room temperature, the mixture was extracted with CH_2_Cl_2_, washed with aqueous NaHCO_3_, and dried over MgSO_4_. The volatiles were removed in vacuo, and the residue was purified by flash column chromatography (SiO_2_) eluted with CH_2_Cl_2_ to give crude tetrahydrothiophene derivative **5** (3.9 g) as a colorless oil:^ 1^H NMR (CDCl_3_) *δ* 2.89 (m, 2H), 2.77 (m, 2H), 2.34 (m, 2H), 1.63 (m, 4H), 1.54 (m, 2H), 1.47 (m, 2H), 1.31 (m, 2H). A toluene solution (60 mL) of the crude **5** and DDQ (8.92 g, 39.3 mmol) was refluxed overnight under nitrogen. The volatiles were removed in vacuo, and the residue was purified by column chromatography (SiO_2_) eluted with hexane to give **6** (1.74 g, 10.4 mmol, 58% from **4**) as a colorless crystal: ^1^H NMR (CDCl_3_) *δ* 6.85 (s, 2H), 3.07 (m, 2H), 1.77 (m, 4H), 1.41 (m, 4H); ^13^C NMR (CDCl_3_) *δ* 145.20, 113.77, 31.08, 26.56; EI-MS: m/z = 164(M^+^). Anal. Calcd for C_10_H_12_S: C, 73.12; H, 7.36. Found: C, 72.96; H, 7.38.

### 4.4. 2,5-dibromo-3,4-bicyclo[2.2.2]octanothiophene *(**7**)*

To a solution of **6** (538 mg, 3.28 mmol) in CHCl_3_ (15 mL) and acetic acid (1 mL), *N*-bromosuccinimide (NBS) (1.24 g, 6.94 mmol) was added in portions and stirred at room temperature overnight. Aqueous NaHCO_3 _was added to the reaction mixture, extracted with CHCl_3_, and dried over MgSO_4_. The volatiles were removed in vacuo, and the residue was purified by column chromatography (SiO_2_) eluted with hexane to give dibromide **7** (1.04 mg, 3.21 mmol, 98%) as a colorless solid. The spectral data were identical with the reported ones [16].

### 4.5. 2-(Trimethylstannyl)-3,4-dioctylthiophene *(**9**)*

A solution of *n*-butyllithium in hexane (1.54 M, 3.00 mL, 4.62 mmol) was added dropwise to a stirred solution of 3,4-dioctylthiophene (**8**) (1.47 g, 4.77 mmol) in THF (50 mL) at –78 °C. After stirring at –78 °C for 1 h, trimethyltin chloride (1.0 M in THF, 4.80 mL, 4.80 mmol) was added, and the reaction mixture was gradually warmed to room temperature over a period of 1 h and stirred at room temperature for an additional 18 h. The crude mixture was washed with aqueous solution of NH_4_Cl, the aqueous layer was extracted with ether, and the ethereal solution was dried over MgSO_4_. The volatiles were removed in vacuo, and the residue was purified by preparative GPC eluted with CHCl_3_ to afford **9** (425 mg, 19%) as a colorless oil: ^1^H NMR (CDCl_3_) *δ* 7.19 (s, 1H), 2.53 (m, 4H), 1.64 (m, 2H), 1.42–1.25 (m, 22H), 0.89 (m, 6H), 0.35 (m, 9H).

### 4.6. 4,5-Bicyclo[2.2.2]octeno-3’,4’-dioctyl-2,2’-bithiophene *(**11**)*

2-Bromo-4,5-bicyclo[2.2.2]octenothiophene (**10**) (219 mg, 0.901 mmol) was mixed with Pd(PPh_3_)_4_ (52 mg, 0.045 mmol), CuO (72 mg, 0.90 mmol) and 5 mL of DMF under argon and the mixture was stirred for 10 min at 100 °C. To this, a solution of **9** (425 mg, 0.902 mmol) in 5 mL of DMF was added and the mixture was stirred at 100 °C for 5 h. The reaction mixture was then allowed to cool down to room temperature, filtered off, and the volatiles were removed in vacuo. The residue was subjected to chromatography on silica gel using hexane to give bithiophene **11** (166 mg, 0.353 mmol, 39%) as a colorless oil: ^1^H NMR (CD_2_Cl_2_) *δ* 6.90 (s, 1H), 6.78 (s, 1H), 3.26 (m, 1H), 3.18 (m, 1H), 2.66 (m, 2H), 2.50 (m, 2H), 1.77 (m, 4H), 1.64 (m, 2H), 1.55 (m, 2H), 1.39–1.28 (m, 16H), 0.88 (m, 6H).

### 4.7. 5’-Bromo-4,5-bicyclo[2.2.2]octeno-3’,4’-dioctyl-2,2’-bithiophene *(**12**)*

To a stirred solution of **11** (166 mg, 0.352 mmol) in 20 mL of carbon tetrachloride at room temperature two drops of 70% HClO_4_ were added, followed by NBS (62.8 mg, 0.353 mmol) in portions, and the mixture was stirred for 12 h. The volatiles were removed in vacuo. The residue was purified by flash chromatography (hexane/SiO_2_) to give **12** (186 mg, 0.338 mmol, 96%) as a colorless oil: ^1^H NMR (CDCl_3_) *δ* 6.85 (s, 1H), 3.26 (m, 1H), 3.18 (m, 1H), 2.65 (m, 2H), 2.51 (m, 2H), 1.76 (m, 4H), 1.52 (m, 4H), 1.41–1.28 (m, 16H), 0.88 (m, 6H); ^13^C NMR (CDCl_3_) *δ* 144.2, 142.3, 140.9, 137.8, 132.2, 130.1, 123.1, 107.2, 31.9, 31.9, 31.2, 31.2, 30.8, 29.7, 29.7, 29.6, 29.3, 29.3, 29.2, 29.2, 28.7, 28.4, 27.3, 26.9, 22.7, 14.2.

### 4.8. 4,5-Bicyclo[2.2.2]octeno-3’’,4’’:3’’’,4’’’-bis(bicyclo[2.2.2]octeno)-3’,4’-dioctyl-2,2’:5’,2’’:5’’,2’’’-quarterthiophene *(**14**)*

A solution of *n*-butyllithium in hexane (1.54 M, 0.42 mL, 0.65 mmol) was added dropwise to a stirred solution of 5-bromo-3,4:3',4'-bis(bicyclo[2.2.2]octano)-2,2'-bithiophene (**13**) (251 mg, 0.619 mmol) in THF (150 mL) at –78 °C. After stirring at –78 °C for 30 min, trimethyltin chloride (1.0 M in THF, 0.65 mL, 0.65 mmol) was added, and the reaction mixture was gradually warmed to room temperature over a period of 1 h and stirred at room temperature for additional 12 h. The crude mixture was washed with aqueous solution of NH_4_Cl, the aqueous layer was extracted with ether, and the ethereal solution was dried over MgSO_4_. The volatiles were removed in vacuo, and the residue was purified by preparative GPC eluted with CHCl_3_ to afford stanylated product (229 mg, 76%) as a colorless solid: ^1^H NMR (CDCl_3_) *δ* 6.81 (s, 1H), 3.33 (m, 1H), 3.31 (m, 1H), 3.04 (m, 1H), 2.95 (m, 1H), 1.81–1.77 (m, 8H), 1.47–1.44 (m, 8H), 0.38 (m, 9H). Bromobithiophene **12** (186 mg, 0.339 mmol) was mixed with Pd(PPh_3_)_4_ (19.6 mg, 0.0170 mmol), CuO (27.0 mg, 0.339 mmol) and 5 mL of DMF under argon and the mixture was stirred for 10 min at 100 °C. To this, a solution of the stanylated product (166 mg, 0.339 mmol) in 50 mL of DMF was added and the mixture was stirred at 100 °C for 2 days. The reaction mixture was then allowed to cool down to room temperature, filtered off, and the volatiles were removed in vacuo. The residue was subjected to preparative GPC eluted with toluene to give **14** (190 mg, 0.239 mmol, 71%) as a yellow solid: ^1^H NMR (CD_2_Cl_2_) *δ* 6.96 (s, 1H), 6.88 (s, 1H), 3.36 (m, 1H), 3.33 (m, 1H), 3.28 (m, 2H), 3.21 (m, 1H) 3.07 (m, 1H), 2.70 (m, 4H), 1.81 (m, 12H) 1.62–1.25 (m, 36H), 0.88 (m, 6H); ^13^C NMR (CD_2_Cl_2_) *δ* 147.0, 145.1, 145.0, 143.0, 142.9, 142.1, 141.3, 139.3, 132.4, 131.3, 127.6, 125.4, 124.7, 124.2, 123.4, 114.2, 32.5, 32.2, 31.9, 31.9, 31.5, 31.4, 31.0, 30.9, 30.8, 30.4, 30.3, 29.8, 29.8, 28.7, 28.6, 27.9, 27.5, 27.0, 26.9, 26.7, 26.7, 23.3, 23.2, 14.5, 14.5; HRMS calcd for C_50_H_66_S_4_, 794.4047; found, 794.4040.

### 4.9. Octithiophene **2**(8T)

Into 10 mL of CH_2_Cl_2_, **14** (68.6 mg, 0.086 mmol) and NO^+^SbF_6_^–^ (23.0 mg, 0.086 mmol) were dissolved with stirring at room temperature. After the resulting dark blue solution was stirred for 24 h, tetrabutylammonium iodide (0.2 g, 0.78 mmol) was added to the solution in one portion. The crude mixture was washed with a saturated aqueous solution of NaHCO_3_ (50 mL), extracted with CH_2_Cl_2_, and dried over MgSO_4_. The volatiles were removed in vacuo and the residue was purified by flash chromatography over SiO_2_ with CH_2_Cl_2_ as an eluent. The eluted product was further purified by preparative GPC eluted with toluene to give** 2**(8T) (57.8 mg, 85%) as a yellow solid; ^1^H NMR (CD_2_Cl_2_) *δ* 6.96 (s, 2H), 3.40 (m, 4H), 3.34 (m, 2H), 3.29 (m, 4H), 3.21 (m, 2H), 1.87–1.73 (m, 24H), 1.61–1.26 (m, 80H), 0.88 (m, 12H); ^13^C NMR (CD_2_Cl_2_) *δ* 145.2, 145.0, 143.8, 143.2, 142.2, 141.3, 139.4, 132.4, 131.3, 127.5, 124.8, 124.7, 124.5, 124.4, 123.4, 32.4, 31.9, 31.9, 31.5, 31.3, 31.1, 31.0, 30.4, 30.2, 29.8, 29.8, 28.7, 28.6, 27.9, 27.5, 26.7, 26.6, 23.3, 23.2, 14.5, 14.5.

### 4.10. Dication salt **2**(8T)^2+^(SbF_6_^–^)_2_

Octithiophene **2**(8T) (47.4 mg, 0.0298 mmol) was dissolved in 1.5 mL of CH_2_Cl_2_ under an argon atmosphere in a 50 mL flask equipped with a glass filter and a rubber septum. To this solution NO^+^SbF_6_^–^ (15.9 mg, 0.0597 mmol) was added in portions and stirred for 30 min. The solution turned deep blue and was filtrated through a membrane filter (0.2 μm). The filtered material was dissolved in dry CH_2_Cl_2_ in a glass tube connected to a vacuum line, and hexane was vapor transferred onto the deep blue solution. Slow diffusion of hexane under an argon atmosphere at –78 °C over 8 days gave crystals of **2**(8T)^2+^(SbF_6_^–^)_2_^–^ (53.8 mg, 87%): Anal. Calcd for C_100_H_130_F_12_S_8_Sb_2_: C, 58.30; H, 6.36. Found: C, 55.86; H, 6.22.
